# Effectiveness of Family Planning Policies: The Abortion Paradox

**DOI:** 10.1371/journal.pone.0091539

**Published:** 2014-03-26

**Authors:** Nathalie Bajos, Mireille Le Guen, Aline Bohet, Henri Panjo, Caroline Moreau, the FECOND group

**Affiliations:** 1 Gender, Sexual and Reproductive Health, CESP Centre for research in Epidemiology and Population Health, U1018, Inserm, F-94807, Le Kremlin-Bicêtre, France; 2 Institut National d’Etudes Démographiques, F-75020, Paris, France; 3 Université Paris Sud, F-94807, Kremlin Bicêtre, France; 4 Department of Population, Family and Reproductive Health, Johns Hopkins Bloomberg School of Public Health , Baltimore, Maryland, United States of America; Indiana University, United States of America

## Abstract

**Objective:**

The relation between levels of contraceptive use and the incidence of induced abortion remains a topic of heated debate. Many of the contradictions are likely due to the fact that abortion is the end point of a process that starts with sexual activity, contraceptive use (or non-use), followed by unwanted pregnancy, a decision to terminate, and access to abortion. Trends in abortion rates reflect changes in each step of this process, and opposing trends may cancel each other out. This paper aims to investigate the roles played by the dissemination of contraception and the evolving norms of motherhood on changes in abortion rates.

**Methods:**

Drawing data from six national probability surveys that explored contraception and pregnancy wantedness in France from 1978 through 2010, we used multivariate linear regression to explore the associations between trends in contraceptive rates and trends in (i) abortion rates, (ii) unwanted pregnancy rates, (iii) and unwanted birth rates, and to determine which of these 3 associations was strongest.

**Findings:**

The association between contraceptive rates and abortion rates over time was weaker than that between contraception rates and unwanted pregnancy rates (p = 0.003). Similarly, the association between contraceptive rates and unwanted birth rates over time was weaker than that between contraceptive rates and unwanted pregnancy rates (p = 0.000).

## Introduction

An understanding of how abortion rates change over time is critical in guiding policies to improve sexual health. As underlined by Marston and Cleland [1], the relation between levels of contraceptive use and the incidence of induced abortion generates heated debate. Some observers argue that abortion rates decrease as contraceptive prevalence increases [2]–[5] while others claim the contrary [1], [6]. Many of the contradictions are likely due to the oversight of the common underlying reason (fertility intentions) for using contraception and undergoing an abortion in case contraceptive strategies fail. A careful understanding of the process leading to abortion is needed to comprehend abortion trends in light of changing contraceptive practices. Indeed, abortion is the end point of a process that starts with sexual activity, followed by the use or non-use of contraception which in turn informs the risk of an unwanted pregnancy. The process continues with the decision to terminate an unwanted pregnancy, and the need to access the health care system for the procedure (where abortion is legal). In this framework, trends in abortion rates reflect changes in each step of the process, with the possibility of opposing trends that statistically cancel each other out [7].

Trend analysis makes it possible to explore the relative contribution of family planning policies on reproductive outcomes over time in the context of changing norms about childbearing. Trend analyses however are considerably rarer [8], [9] than multi-country cross-sectional analyses [1], [7], [10] in developed countries, mostly because of the lack of reliable data covering long periods of time. The validity of comparisons using different research instruments raises questions, however, as differences may reflect true changes or differences in survey methodology [11]. Few countries have the luxury of large and comparable cohort studies or repeat national cross-sectional surveys that investigate contraceptive practices, pregnancy wantedness, and abortion practices in the context of widespread access to contraception.

French data provide such a historical perspective with comparable data available from the mid-1970s onward. Thus it is possible to examine trends in contraceptive behaviors and their association with reproductive health outcomes in the context of continuous support for family planning since the legislation of contraception (in 1967) and abortion (in 1975). In France, policies have consistently encouraged use of contraception by providing information through sexual education and national contraception campaigns since the early 1980s, and facilitating access through publicly funded family planning clinics and offering coverage of the cost by the national health insurance system. The law has also continuously ensured access to abortion, partly reimbursed by the national health insurance system since 1981 (and totally covered since 2013).

This paper presents a retrospective analysis of contraceptive behaviors, pregnancy intentions, and recourse to abortion over four decades. We investigate the roles played by the dissemination of contraception and the evolving norms of motherhood on changes in abortion rates and question the validity of abortion rates as an accurate indicator for monitoring family planning policies.

At a time when the effectiveness of family planning policies is being questioned, this socio-historical perspective provides us with a unique opportunity to contribute to this important scientific and public health debate.

## Materials and Methods

### Study Design

Data come from six national probability surveys that explored lifetime contraceptive use and pregnancy intentions in France from 1978 through 2010 (Table 1) and from annual national statistics on abortions. All surveys studied national probability samples of several thousand women (range: 1,431 to 5,272) aged 18 to 44 years (the lowest age in the 1978 and 1994 surveys was 20). Sampling design and post-stratification weights were applied, using the same methodology in all surveys. Data are from national surveys and demographic statistics, so available upon request.

**Table 1:** Methodological characteristics of the 6 national surveys on contraceptive and reproductive behaviors in France.

	1978	1988	1994	1998	2000	2010
Sample size	2 982	3 183	2 944	1 431	2 863	5 272
Age range	20–44	18–49	20–49	18–45	18–44	15–49
Sampling design	random	random	random	random	random	random
Variables	contraceptive use	contraceptive use	contraceptive use		contraceptive use	contraceptive use
	wantedness of each pregnancy	wantedness of each pregnancy	wantedness of each pregnancy	wantedness of each pregnancy	plannedness* of each pregnancy	wantedness of each pregnancy
Mode of administration	Face to face	Face to face	Face to face	Face to face	Telephone	Telephone
Response rate	85%	79%	84%	94%**	77%	74%
Post stratification	Age, region, occupational status, marital status	Age, region, occupational status, marital status	Age, region, occupational status, marital status	Age, region, occupational status, marital status	Age, region, occupational status, marital status	Age, region, occupational status, marital status

*Women were asked if they had *planned* to be pregnant in the 2000 survey whereas they were asked if they *wanted* to be pregnant in the other surveys.

**The survey was offered to a national random sample who answered a mandatory governmental survey.

### Outcomes

#### Contraceptive practices

We present the distribution of contraceptive practices in 1978, 1988, 1994, 2000, and 2010 (Table 2). In case of dual use, we retained the most effective method, based on typical use failure rates published in the literature [12]. As 18- and 19-year-olds were not interviewed in 1978 and 1994, we used retrospective information collected in women’s lifetime contraceptive histories in the 1988 survey (among those aged 28 and 29 years) and in the 2000 survey (among those aged 24 and 25 years) to reconstruct their use of contraception in 1978 and 1994.

**Table 2:** Proportion of women 18–44 years old using selected contraceptive methods, by year.

	1978		1988		1994		2000		2010	
	%	95%CI	%	95%CI	%	95%CI	%	95%CI	%	95%CI
Sterilization	-		5.4	4.5–6.4	-		4.4	3.4–5.6	2.9	2.3–3.6
Pill	27.2	25.6–28.7	34.5	32.7–36.4	40.4	38.5–42.2	46.0	43.4–48.6	41.3	39.5–43.0
IUD	7.7	6.8–8.6	17.5	16.2–19.0	15.0	13.7–16.3	16.1	14.3–18.0	14.1	13.0–15.3
Implant, patch, injections, vaginal ring	0.0		0.0		0.0		0.0		3.5	2.9–4.2
*Very effective reversible methods* *	*34.9*	*33.2*–*36.5*	*52.1*	*50.1*–*54.0*	*55.4*	*53,0*–,*57.3*	*61.9*	*59.6*–*64.6*	*58.9*	*57.1*–*60.6*
Condom	-		3.3	2.7–4.0	4.8	4.0–5.6	7.5	6.3–9.0	8.6	7.6–9.6
Withdrawal	17.1	15.8–18.4	4.5	3.8–5.4	2.2	1.7–2.8	2.1	1.4,3.1	2.7	2.2–3.3
Others (rhythm, cervical cap…)	12.4	11.2–13.5	6,6	5.7–7.6	2.7	2.1–3.3	2.1	1.5–2.8	2.1	1.7–2.8
No contraception	-		4.4	3.4–5.2	-		2.7	2.0–.3.6	2.2	1.6–2.8
Not concerned (sterile, pregnant, wants to become pregnant)	-		11.7	10.5–13.0	-		9.1	7.8–10.5	11.6	10.5–12.8
No partner			12.2	10.8–13.7			10.0	8.4,11.9	11.0	9.9–12.2
Total			100		100		100		100	

*Pill, IUD, Implant, Patch, Injections, vaginal ring.

All percentages are weighted to account for sampling design and post-stratification.

To calculate annual contraceptive rates for women aged 18–44, we applied age-specific rates for each survey year to the age distribution of the female population in France during those years.

#### Unwanted pregnancy rates

In each survey, women completed a lifetime pregnancy history. We classified each pregnancy as “*wanted*” if women stated they had wished to become pregnant “at that time” or “earlier”, and “*unwanted*” if they had wished to become pregnant “later” or “not at all” or if they stated they had “not thought about it”.

Women who did not answer this question, *i.e.* between 0.5% and 3.2% of the women in the selected surveys, were excluded from the analysis.

As abortions are underreported in population-based surveys [8], [13], we cannot use survey data to estimate unwanted pregnancies ending in abortion. Therefore, we estimated only *unwanted births* from the population-based surveys. We applied the proportion of wanted and unwanted births estimated from each survey to the national fertility rates (derived from national birth statistics) of the corresponding period to estimate national wanted and unwanted birth rates. National abortion rates (based on mandatory reporting of abortions in France) were then added to calculate the overall unwanted pregnancy rate as the sum of national unwanted birth and abortion rates (assuming all abortions were unwanted pregnancies). The probability of terminating an unwanted pregnancy was defined as the abortion rate divided by the unwanted pregnancy rate.

To have a sufficient number of events to produce robust indicators, we estimated the proportion of births that were unwanted at the time of each survey as the average proportion of births that were unwanted over the 5-years period preceding each survey. We thus present unwanted pregnancy rates for the periods 1973–77, 1978–82, 1983–87, 1988–92, 1993–97, 1998–2002 and 2005-2009 (Table 3). Abortion rates represent the annual abortion rate of the median year of each interval.

**Table 3:** Estimates of lifetime pregnancy rates by year, age group, and pregnancy intentions.

	survey year	1978	1988	1988	1994	1998	2010	2010
	Period	1973–77	1978–82	1983–87	1988–92	1993–97	1998–2002	2005–09
Wanted birth (1)	18–29 y	1.01	1.18	1.09	0.98	0.90	0.90	0.91
	30–44 y	0.28	0.32	0.36	0.44	0.52	0.62	0.69
	18–44 y	1.29	1.50	1.45	1.42	1.42	1.52	1.60
Unwanted birth (2)	18–29 y	0.45	0.27	0.23	0.22	0.18	0.20	0.20
	30–44 y	0.14	0.09	0.09	0.10	0.09	0.10	0.16
	18–44 y	0.59	0.35	0.33	0.32	0.28	0.30	0.36
Total fertility rate (1+2)	18–29 y	1.46	145	1.32	1.20	1.08	1.10	1.11
	30–44 y	0.42	0.41	0.45	0.54	0.61	0.72	0.85
	18–44 y	1.88	1.86	1.77	1.74	1.69	1.82	1.96
Abortion (3)	18–29 y	0.36	0.34	0.30	0.27	0.27	0.29	0.30
	30–44 y	0.27	0.25	0.22	0.20	0.19	0.19	0.19
	18–44 y	0.64	0.59	0.52	0.47	0.46	0.48	0.49
Unwanted pregnancies (2+3)	18–29 y	0.82	0.61	0.53	0.49	0.46	0.49	0.49
	30–44 y	0.41	0.33	0.31	0.29	0.28	0.29	0.35
	18–44 y	1.23	0.94	0.84	0.78	0.74	0.78	0.85
All pregnancies*(1+2+3)	18–29 y	1.82	1.78	1.62	1.47	1.36	1.39	1.40
	30–44 y	0.70	0.66	0.67	0.73	0.80	0.91	1.05
	18–44 y	2.52	2.44	2.30	2.20	2.15	2.31	2.45
% of unwanted pregnanies ended in abortion (3/(2+3))	18–29 y	44	56	56	55	59	59	60
	30–44 y	66	74	70	67	68	66	54
	18–44 y	52	63	61	59	63	61	58
Age at first birth (4)		23.9	24.4	25.3	26.0	26.8	27.4	27.9
Ideal number of children (5)		2.5	2.5	2.4	2.5	2.4	2.4	2.4

Results indicate that in 1973–77 women would have had 2.52 lifetime pregnancies according to the fertility age rates observed at that period. Among these pregnancies, 1.29 would have been wanted births, 1.23 unwanted pregnancies (0.59 unwanted births and 0.64 abortions).

* Miscarriages are not included.

(1) (2) Surveys data.

(3) National statistcs.

(4)National statistics (Pison 2010).

(5) Surveys data (Beaujouan and Toulemon. 2013) for women aged 25–34.

Finally, we also present data on age at first birth based on national statistics [14] and on ideal family size based on national survey data [15].

We present the results for all women and for two age groups: 18–29 years and 30–44 years.

To ensure that the social and demographic composition of the French female population for each survey year was accurately portrayed, sampling and post-stratification weights were used in each survey data set to take into account the complex survey designs and sampling distortions [16].

### Statistical Analysis

#### Estimation of rates over time

To estimate rates of contraceptive use and unwanted pregnancy for the same years, we used linear regression to model contraceptive use, known at the time of the surveys (from 1978 to 2010), and unwanted pregnancy rates, known for the median year of the period for each of the 5-years periods (from 1973–77 to 2005–09). We thus obtained predictive values of effective contraceptive prevalence rates and unwanted pregnancy rates for the following ten years: 1975, 1978, 1980, 1985, 1988, 1990, 1994, 1995, 2000, 2007 (Table 3). Abortion rates were available from national statistics for each year.

#### Associations between trends

To test if the association between trends in effective contraceptive prevalence rates and unwanted pregnancy rates was stronger than the association between effective contraceptive prevalence rates and abortion rates, we explored the association between trends in effective contraceptive prevalence rates and trends in *(i)* abortion rates, *(ii)* unwanted pregnancy rates, and *(iii)* unwanted birth rates and used a multivariate linear regression [17] with three dependent variables (abortion, unwanted pregnancy and unwanted birth) to determine which of these three associations was strongest (Table 4).

**Table 4:** Associations between contraceptives prevalence rates and abortion, unwanted pregnancy, and unwanted birth rates from 1973–77 to 2005–09.

		18–44	18–29	30–44
(1) Contraception and abortion	β	–0.2	–0.3	–0.2
	95%CI	–0.3–0.1	–0.3–0.3	–0.2–0.1
(2) Contraception and unwanted pregnancy	β	–0.5	–0.9	–0.2
	95%CI	–0.7. –0.3	–1.2–0.6	–0.3–0.1
(3) Contraception and unwanted birth	β	–0.3	–0.6	–0.01
	95%CI	–0.4–0.1	–0.9–0.4	–0.1–0.1
p (1)/(2)		0.003	0.001	0.872
p (2)/(3)		0.00	0.00	0.00

Results from multivariate linear regressions.

All analyses were performed with Stata statistical software (version 12.1).

### Ethical issues

All national surveys were approved for ethical and confidentiality issues by the French national data protection agency, the “Commission Nationale Informatiques et Libertés”.

## Results

### Contraceptive use

These results underline the dramatic increase in the use of very effective methods of contraception (pill and IUD in France) over the last four decades. In the last 4 decades, the proportion of sexually active women has remained stable. During the same period, the proportion of women at risk of an unwanted pregnancy (*i.e.,* who are sexually active and not pregnant or trying to become pregnant or sterile) who rely on very effective methods has regularly increased, from 35% of the 18–44-year age group in 1978 to 52% in 1988 and 62% in 2000 (Table 2). A slight decrease was observed for the first time between 2000 and 2010 (from 62% to 60%).

Only a small minority of women at risk of an unwanted pregnancy (*i.e.,* who are sexually active and not pregnant or trying to become pregnant or sterile) do not use any form of it, and this proportion has decreased over time, from 8.2% in 1978 to 2.2% in 2010.

### Pregnancy wantedness

The dissemination of very effective methods of contraception has resulted in a regular decrease in unwanted pregnancy rates until the mid-1990s (from 1.23 unwanted pregnancy per woman aged 18–44 in 1973–1977 to 0.74 in 1993–1997 for the same age group) (Table 3). Over the past decade, unwanted pregnancy rates have increased slightly, reaching 0.85 per woman in 2005–2009 (Table 3).

Unwanted pregnancy rates have dropped more substantially among young women than among their older counterparts. The 40% decline observed between 1973–77 and 2005–09 among women less than 30 years old (from 0.82 to 0.49 unwanted pregnancies per woman) was most marked for unwanted pregnancies leading to births (56% decline). Wanted birth rates decreased slightly during the same period (from 1.01 to 0.91 wanted births per woman aged 18–29 years). Among women 30 years or older, the unwanted pregnancy rate decreased by 31% from 1973–77 to 1993–97 (from 0.41 to 0.28 unwanted pregnancy per woman) and has risen since (to 0.35 in 2005–09). On the other hand, the rate of wanted births among women 30 years and older has increased substantially (from 0.28 to 0.69).

### Termination of unwanted pregnancies

**Figure 1:**
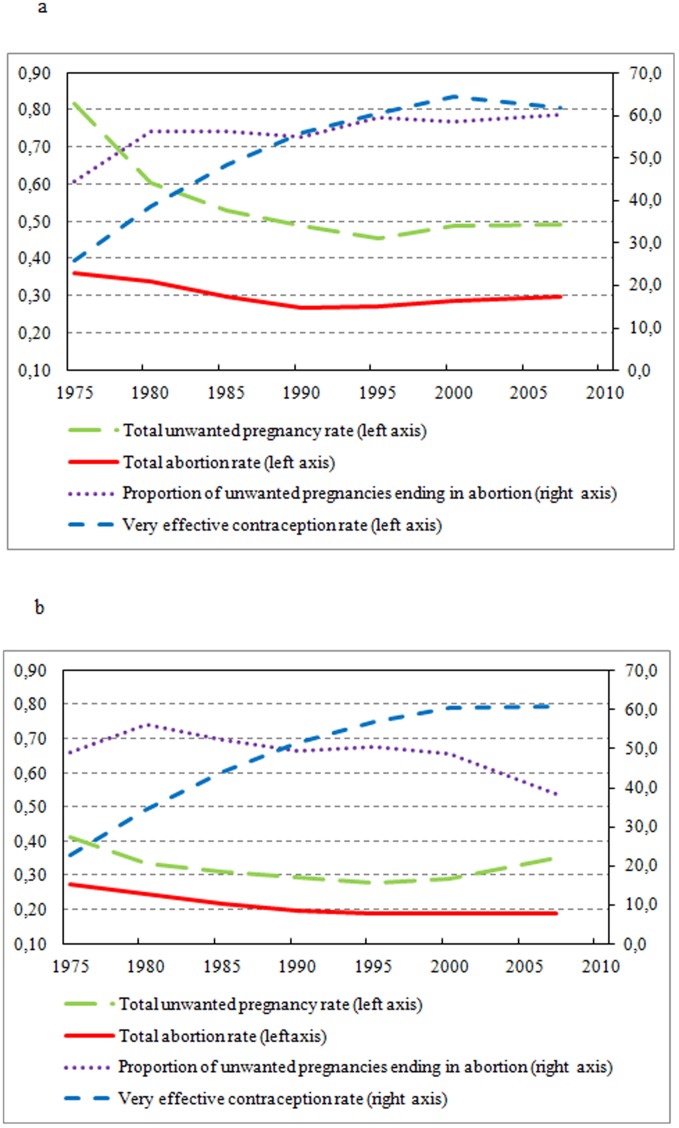
Trends in unwanted pregnancies, probability of ending them, and abortion rates. A) for women 18–29, and B) for women 30–44.

Among women younger than 30 years, the probability of ending an unwanted pregnancy increased from 44% in 1973–77 to 59% in 1993–97 and has remained stable in this age group ever since (Figure 1a). Thus the trend in abortion rates until the late 1990s reflects only partially the impact of effective contraception, as a growing proportion of unwanted pregnancies ended in abortions (Table 3).

The reverse was observed among women 30 years or older (Figure 1b): after increasing from 1973–77 to 1978–82 (from 66% to 74%), the probability of ending an unwanted pregnancy fell to 54% in 2005–09 (Table 3).

Over the past 4 decades, age at first birth has consistently increased from 23,9 years old in 1978 to 27.9 years old in 2010. Over the same period, the ideal number of children has remained stable for women aged 25–34 years old: 2.4 children.

### Contraception, unwanted pregnancies, and abortion

Results from the multivariate linear regression (Table 4) show that the association between rates of very effective contraceptive use and of abortion from 1975 through 2007 is weaker than the association between very effective contraceptive rates and unwanted pregnancy rates (p = 0.003). In other words, a 1 percentage point increase in the very effective contraceptive rate yields a 0.2 per 1000 decrease in the abortion rate; it yields a 0.5 per 1000 decrease in the unwanted pregnancy rate. The difference in the association between trends in very effective contraceptive rates and abortion rates on the one hand and the association between trends in very effective contraceptive rates and unwanted pregnancy rates on the other hand was significant among women younger than 30 years (p = 0.001), but not among older women (p = 0.872).

In addition, the association between trends in very effective contraceptive rates and unwanted birth rates for all women was weaker than the association between very effective contraceptive rates and unwanted pregnancy rates (p = 0.000); the same was observed for both age groups.

Finally, abortions rates were not significantly associated with birth rates (r = +0.68; p = 0.09, IC95% [–0.14–1.49]).

## Discussion

These results drawn from serially conducted national fertility surveys underline the dramatic diffusion of very effective contraception methods over the past four decades in France. These changes in contraceptive behavior are associated with significant decreases in rates of both unwanted pregnancies and abortions. The decline in unwanted pregnancy rates was greatest among women younger than 30 years old. Our results also indicate that effective contraception uptake was more strongly linked to unwanted pregnancy rates than to unwanted birth rates or abortion rates.

The study has limitations that we now discuss.

The challenges of comparative analyses, well documented in cross-national comparisons [11], equally apply to time-series analyses. The French fertility surveys used the same survey instrument and generally followed the same research design (national household probability samples), which limited methodological differences in measurement. The last two surveys were conducted by telephone instead of face-to-face. Nevertheless, previous research has shown that mode of survey administration (telephone *versus* face to face) has no effect on responses to sensitive questions on sexual and reproductive practices in France [11]. Response rates have declined over time, which may have affected the validity of the comparison. For this reason, each survey was weighted to reflect the social and demographic composition of the French population at that time. Moreover our estimators of very effective contraceptive use matched sales figures for contraceptive agents, as did our estimates of birth rates compared with those based on the national birth registry (data not shown).

Independent of survey methods, the meaning of pregnancy wantedness may be particularly sensitive to contextual influences [18]. Therefore, fluctuations in unwanted pregnancy rates may result both from improved contraceptive effectiveness and from changes in the social conceptualization of unwanted pregnancy. In any event, the objective of family planning policies is to help women and men prevent unwanted pregnancies, however they may be defined, by using contraception.

Our estimates of unwanted pregnancy rates rest solely on estimates of unwanted births and abortions and assume that all pregnancies that ended in abortion were unwanted, which appeared to be the case in more than 95% of the abortions reported in these surveys (data not shown). We did not include pregnancies resulting in miscarriages or other outcomes in the calculation, because they are either poorly reported in population-based surveys or very infrequent. We assumed that the proportion of unwanted pregnancies for these pregnancy outcomes was the same as for all other pregnancies. We also assumed that our samples have the same abortion history as the whole population.

Finally, we hypothesized that effective contraceptive use and unwanted pregnancy rates changed linearly between two survey points (on average, 5 years apart). We have no external data to confirm or refute this hypothesis (except for pill use provided by sales figures). Nevertheless, there is no reason to think that the correlation between the two variables would change, even if the rate changes were not linear.

Despite these limitations, our analysis adds important information to the ongoing debate about the impact of contraception on unwanted pregnancies and abortion. Research conducted in the US and Europe has generally concluded that abortion rates are higher where contraceptive availability is limited [3], [5]. The link is less clear in countries with low contraceptive prevalence [1], [10], [19], [20]. Many of the latter are undergoing radical demographic transitions, and the desire for smaller families drives increases in both contraceptive uptake and abortion.

Consistent with prior studies in the US [21], our results show no association between time trends in either contraceptive or abortion rates and time trends in the proportion of sexually active women. Thus both age at first intercourse in France and the proportion of sexually active women have remained stable over time [22] while contraceptive use has substantially increased. While we have no information regarding the proportion of women who intentionally engage in unprotected sexual activity, an issue explored in other studies [23], our results suggest that this phenomenon is rare as less than 3% of women do not use contraception while at potential risk of an unintended pregnancy. This proportion has remained stable over time. Our findings also indicate that a high abortion rate is not correlated with a low birth rate and *vice versa* which suggests that an abortion does not avert a birth.

Our results highlight the importance of considering abortion rates as not only a result of contraceptive effectiveness but also a reflection of changing social norms about childbearing. This can be seen in the growing contrast between younger and older women as the propensity to terminate unwanted pregnancies evolves. With the continuing postponement of childbearing over the last four decades (first birth occurs at 27.9 years old in 2010 compared to 23.9 years old in 1978), young women are more likely to end an unwanted pregnancy today than they were in the past while their older counterparts have become less likely to do so in recent years. These changing patterns in pregnancy terminations have taken place in the context of significant social changes in women’s status, characterized by sharp increases in school enrolment and labor force participation. These developments were particularly manifest in the 1970s in the aftermath of the legalization of abortion, a time when the propensity to end an unwanted pregnancy rose sharply. The evolution of women’s sexual and relationship trajectories towards greater diversification, paralleling these socioeconomic changes, has influenced pregnancy intentions and decisions to terminate unwanted pregnancies and continues to do so [24]–[26].

Thus, the dissemination of effective contraception and increased recourse to abortion have accompanied a paradigm shift towards older elective parenthood, with no impact on the ideal family size which remains stable at around 2.5 children. Children are to be “planned” in a stable emotional context, and at the “right” time in the parents’ educational and professional career paths [27]. These changing social expectations, along with rising socioeconomic instability, which has affected young women in particular, are likely to explain the differences in associations between contraceptive effectiveness and abortion rates between young and older women. While very effective contraception affected unwanted birth rates more than abortion rates among young women, the reverse was true among older women, who are more likely to meet the socially accepted socioeconomic and emotional conditions for parenthood.

Ultimately, changes in the rate of abortions of unintended pregnancies reflect changes in the procreative norm, which goes far beyond health policies.

## Conclusion

Trends in use of effective contraceptive methods are associated with reductions in unwanted pregnancy rates. But the probability of ending an unwanted pregnancy increased over time among the youngest women whilst it decreased among older women. Abortion rates result from a complex process that depends not only on contraceptive coverage and effectiveness but also on the changing procreative norms, which inform the decision to end an unwanted pregnancy. Thus, unwanted pregnancy rates rather than abortion rates should be considered for evaluating family planning policies.
